# Using Patient-Reported Outcome Measures to Promote Patient-Centered Practice: Building Capacity Among Pediatric Physiotherapists in Rwanda

**DOI:** 10.9745/GHSP-D-19-00408

**Published:** 2020-09-30

**Authors:** Monika Mann, Ines Musabyemariya, Linn Harding, Ben Braxley

**Affiliations:** a Department of International Health, Johns Hopkins Bloomberg School of Public Health, Baltimore, MD, USA.; b Humanity and Inclusion, Kigali, Rwanda.; c West County Hand and Physical Therapy, Sebastopol, CA, USA.; d Dignity Health, Fair Oaks, CA, USA.

## Abstract

**Background:**

Patient-reported outcome measures (PROMs) assess disability and progress toward functional goals while promoting patient-centered practice. They can be used by health professionals in any specialty and in a multitude of settings. This study reviews implementation strategies and lessons learned in a capacity-building program that took place with pediatric physiotherapists in Rwanda.

**Methods:**

Use of PROMs and patient-centered practice were integrated into 4 consecutive continuing professional development courses offered to 164 participants in Rwanda. We sought to identify a simple generic measure with proven validity in cross-cultural settings. The Patient-Specific Functional Scale was chosen due to its ease of use and ability to measure change in a wide range of patient conditions. Didactic classroom training and clinical site visits were 2 essential pedagogical elements of the capacity-building strategy. Site visits allowed for evaluation of skill levels and facilitation of knowledge transfer to patient care settings. Unique pairs of Rwandan colleagues were trained to serve as coteachers in each course to maximize sustainability of new techniques. This study presents data on a subset of 65 participants who completed a 48-hour pediatric rehabilitation course.

**Results:**

After classroom instruction, 78% of participants were observed independently determining functional limitations with their patients. Additionally, pre- and post-tests indicated that therapists substantially increased their understanding of patient-centered practice after attending courses. Interviews conducted 26 months after the conclusion of the project revealed mixed success in sustainability of the use of PROMs, although perceived confidence remained high.

**Conclusion:**

Challenges in long-term sustainability of new practices call attention to the need to target not only clinicians when introducing new methodologies, but also the Ministry of Health, hospital administration, and university faculty. Lessons learned from this study may be useful to other medical professionals planning capacity-building programs in low- and middle-income countries.

## Abstract (precis)

Tracking outcomes is integral to assessing effectiveness of health systems. Multimodal training was offered in the use of a contextually appropriate, patient-centered outcome measure in a low-resource setting. Results offer insights for designing future capacity-building programs.

## Teaser

**Key Findings:**

After completing the training:

- Ninety-two percent of respondents stated that they felt either “quite” or “very” confident in their abilities to establish functional goals.
- Seventy percent of respondents stated that they continue to use patient-reported outcome measures either “a lot” or “quite a bit” 26 months after the close of the grant.

**Key Implications:**

Program managers should consider:

- Targeting stakeholders from a range of health system levels to facilitate systemic and institutional support of practice changes as well as maximize adoption, penetration, and sustainability of new skills and concepts
- Adopting culturally appropriate outcome measure tools
- Using multimodal strategies for capacity building

## INTRODUCTION

**FIGURE 1.**
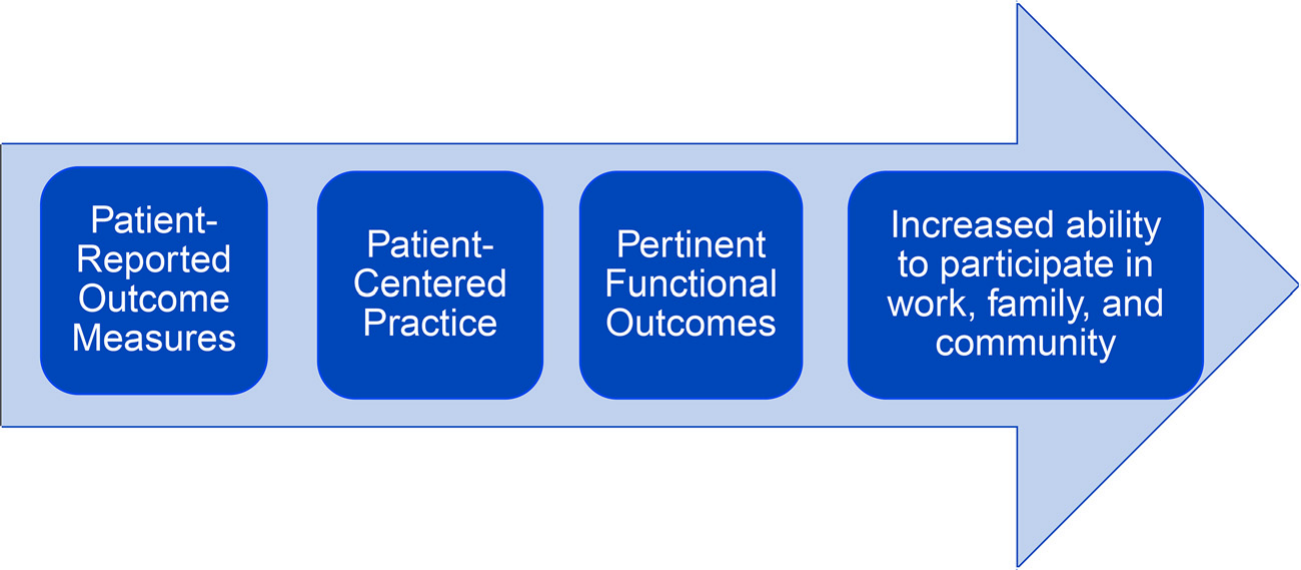
Patient-Reported Outcome Measures

Tracking health care outcomes is integral to assessing effectiveness and efficiency within health systems. Historically, the data have been based on medical tests of body function and structure^1^ performed by a clinician. Patient-reported outcome measures (PROMs) were developed to focus on activity limitations and participation restrictions reported by the patient (Figure 1).^2^^,^^3^ Treatment planning and assessment can thereby focus on the outcome goals of the patient rather than solely the clinician’s objectives. PROMs not only take into account the patient’s experience, but also tend to be oriented toward quality-of-life measures including function and ability to participate in society.^1^

Standardized and validated instruments to measure outcomes are utilized to both guide treatment and determine its functional impact.^3^ It is crucial to integrate PROMs as components of the data routinely collected and analyzed to promote patient-centered practice.^4^ Although PROMs can be used by health professionals in any specialty and in a multitude of settings, this report reviews implementation strategies and lessons learned during a capacity-building program to increase the use of PROMs and promote patient-centered practice among pediatric physiotherapists in a low-resource country.

## PROGRAM DESCRIPTION

The Advancement of Rwandan Rehabilitation Services Project (ARRSP) was a 27-month program funded by the United States Agency for International Development and implemented by Health Volunteers Overseas from March 2013 until May 2015. Key goals of the program were to upgrade rehabilitation standards in Rwanda and improve the quality of services provided. This aim was in alignment with Rwanda’s Third Health Sector Strategic Plan, July 2012–June 2018, in which one of the strategies listed was to “train health workers on control, prevention, and treatment of injuries and disabilities.”^5^

Key goals of the ARRSP were to upgrade rehabilitation standards in Rwanda and improve the quality of services provided.

The ARRSP offered successive continuing professional development courses to practicing physiotherapists. Content specialists were recruited from the United States to teach a series of courses on topics that had been selected by a steering committee made up of Rwandan rehabilitation professionals. The committee also chose 2 different Rwandan physiotherapists to be co-instructors for each course. Preference was given to physiotherapy faculty members so that they could integrate new concepts and practices into their teaching of students at the university. During the grant period, there were 4 physiotherapy faculty members in the country, and all participated in the courses. Rwandan co-instructors received intensive training on PROMs, course content, and patient-centered practice. This study focuses on the participants of the pediatric rehabilitation course since a follow-up assessment was conducted on this subgroup 26 months after the conclusion of the grant to determine sustainability of introduced skills and concepts.

Courses ranged from 36 to 48 hours in length and were offered to 2 or 3 cohorts at a frequency of one weekend class session per month per cohort. By the last class session each month, Rwandan co-instructors were responsible for the majority of teaching. At the time the courses were given, records from the Rwanda Allied Health Professionals Council indicated that there were 142 registered physiotherapists employed in direct patient care in the country. One hundred sixty-four therapists attended at least one of the courses included in this study, with most participants attending 2 or 3 continuing professional development offerings (Table 1). This indicates that virtually all practicing physiotherapists as well as some therapists who did not work directly in patient care attended at least one of the multi-weekend offerings. About 22 physiotherapists worked with a population that was at least 75% pediatric. Most other therapists were generalists and saw a mix of adult and pediatric patients.

**TABLE 1.** Data on Continuing Professional Development Courses Offered as Part of the Advancement of Rwandan Rehabilitation Services Project That Emphasized Patient-Specific Outcome Measures and Patient-Centered Practice

**Course Name**	**Cohort Groups**	**Classroom Hours per Cohort**	**Class Sessions per Cohort**	**Number of Participants**
Therapeutic exercise	3	48	4	81
Neurological rehabilitation	3	48	4	90
Pediatric rehabilitation	2	48	4	65
Leadership institute	2	36	3	69

All courses incorporated didactic classroom teaching as well as training in clinical settings. An essential element in both environments was the use of PROMs and patient-centered clinical decision making to facilitate patients’ return to optimal function. To achieve this, we set out to select a contextually appropriate, generic PROM that could be used across all courses.

### Outcome Measure Selection

Since the use of outcome measures has been identified as an essential component of best practices in physical therapy,^6^^,^^7^ an objective of the ARRSP was to instruct Rwandan physiotherapists in the use of an appropriate PROM that would promote patient-centered practice. The use of outcome measures was a new concept among the majority of physiotherapists in Rwanda, so we sought to identify a simple, generic measure that could be used across multiple diagnoses. Both the World Health Organization Disability Assessment Schedule 2.0 (WHODAS 2.0) and Patient-Specific Functional Scale (PSFS) have been shown to be valid in cross-cultural settings and can be used to measure change in a wide range of patient conditions.^8^^–^^13^ Therefore, these were the 2 PROMs considered for use.

The use of outcome measures was a new concept among the majority of physiotherapists in Rwanda.

**FIGURE 2.**
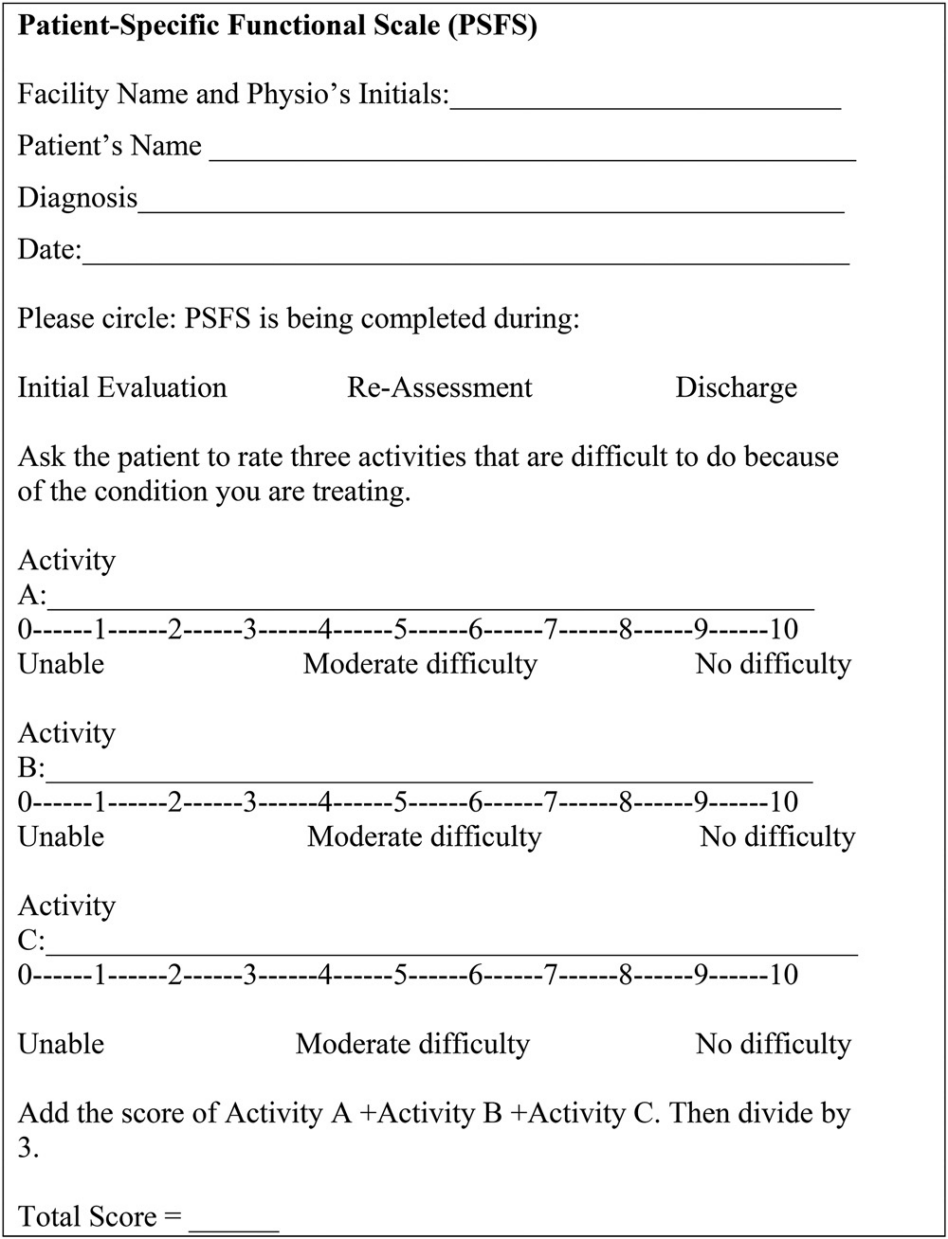
Patient-Specific Functional Scale

Studies have demonstrated that consideration of local, contextual factors is often essential to the long-term success of a project.^14^^,^^15^ This is especially salient when working outside of one’s usual environment. During our early meetings with University of Rwanda faculty and administration, we were advised that the Rwandan culture tended toward oral communication and that processes involving extensive reading or writing could represent barriers to the acceptability, adoption, and sustainability^16^ of a PROM. Additionally, in many clinics and hospitals, the resources necessary to produce a lengthy PROM, such as the WHODAS 2.0, were not reliably available. This barrier cast doubt on the appropriateness and feasibility of the WHODAS 2.0 for use in everyday clinical practice. The PSFS can be administered verbally with little reading required, and minimal space is needed to record scores (Figure 2).

An additional strength of the PSFS is its inherent patient-centeredness. Rather than using predefined activities that might lack a local context, the PSFS calls on patients to identify and assign a rating to activities that they have difficulty completing as a result of their impairment. In this manner, the scale is tailored toward activities that are relevant to each patient and a separate pediatric version is not necessary. For infants, toddlers, and nonverbal children, functional deficits and goals can be determined by a proxy, such as parents. The measure has demonstrated reliability and validity for use in impairments of the lower extremity, upper extremity, and the cervical and lumbar spine.^17^ Additionally, the use of PROMs in a pediatric population has been described by a number of authors.^18^^–^^21^ Taking these attributes into consideration, we selected the PSFS for use throughout the ARRSP.

### Capacity-Building Strategies

The ARRSP sought to build capacity through training clinicians and rehabilitation faculty in PROMs and patient-centered practice. Once we determined that the PSFS would be the PROM of choice, the course instructors and project coordinator began an iterative process of refining implementation techniques for capacity building in both use of the measure and improvement of patient-centered practice. Because 4 unique instructor teams each taught successive courses covering a different topic in rehabilitation, capacity building evolved as the unique needs of each cohort became apparent. A 3-pronged approach was used to maximize acceptance, adoption, penetration, feasibility, and sustainability of the measure.

The ARRSP sought to build capacity through training clinicians and rehabilitation faculty in PROMs and patient-centered practice.

### Three-Pronged Approach to Capacity Building

#### 1. Modeling and Reinforcing PSFS Technique and Patient-Centered Practice

Course instructors used the World Health Organization’s International Classification of Functioning, Disability and Health (ICF)^2^ as a model for teaching and reinforcing the concept of functioning and functional goals. Classroom time was scheduled to allow for multiple repetitions of modeling appropriate PSFS utilization. Didactic teaching included lecture, case study discussions, role-playing, and hands-on skills training. Emphasis was placed on using results of the PROM to design patient-centered treatment plans and objectively tracking progress toward functional goals. Classroom work in small groups allowed for discussion on how to revise treatment plans based on changes in PSFS scores during reassessments. Instructors often referenced cases observed during clinic visits to provide culturally appropriate examples of PSFS use and relevance to patient-centered practice.

#### 2. Distributing a Standardized Booklet for Ease of Use by First-Generation PROM Adopters

In an attempt to maximize adoption and feasibility of the PSFS, a booklet was drafted and distributed to participants that included instructions and rationale for its use. The booklet also contained a completed sample PSFS and 3 blank forms. PSFS data collected via site visits and booklets were presented in the classroom to facilitate discussions on how results can be used to assess and progress treatment.

#### 3. Evaluating the Use of the PSFS and Patient-Centered Practice During Clinical Visits

The inclusion of clinical site visits was an integral component of the ARRSP and was thought to be essential for achieving maximal adoption and penetration of the PSFS and patient-centered practice. Site visits provided an opportunity to offer direct clinical mentoring and observation. This allowed instructors to evaluate skill levels and reinforce classroom lessons to help facilitate knowledge transfer to patient care settings. A site visit checklist was utilized to evaluate clinical reasoning, documentation of functional problems, patient-centered practices, and implementation of the PSFS. Formative feedback was offered to those who needed assistance completing the measure or had difficulty relating it to patient-centered practice. Course participants typically invited colleagues to engage in clinical visits so that the number of rehabilitation professionals influenced by the visits substantially exceeded the number formally attending classes.

## RESULTS

We used 6 measures to assess the success of the project:

1. Pre- and post-tests for each course
2. Clinical observations
3. Participants’ written assessments of each course
4. End-of-grant written assessments
5. End-of-grant interviews with course participants
6. Interviews and survey conducted 26 months after the conclusion of the ARRSP

Six measures were used to assess the success of the project, including an evaluation 26 months after the grant ended.

### Pre- and Post-tests

Pre- and post-tests were a part of every course. Item analysis of the pre- and post-tests indicated that participants’ understanding of PROMs and patient-centered practice increased after attending courses. An example of a question included in the pre- and post-test of the pediatric rehabilitation course is shown (Box). Supplement 1 contains the full pre- and post-test questionnaire. On the pretest, 55% of the participants (N=66) selected option B, the correct answer. On the post-test, 94% (N=61) chose the correct answer, demonstrating an increased understanding of the value of the PSFS.

BOX. Example of Pretest/Post-test Pediatric Rehabilitation Course Question

Using the Patient Specific Functional Scale (PSFS) with our pediatric patients and their families can help us:

- Perform a standardized, norm-based assessment allowing us to compare our patient to other children his/her age.
- Understand and identify what activities are important to the child and his/her family.
- Make a diagnosis about what is wrong with the child.
- The information we learn from the PSFS does not really help us in our evaluation, choice of treatment or setting of goals for the child.

### Clinical Observations

Course instructors evaluated the participants’ ability to identify functional deficits during structured clinical observations. This skill was new and was not previously a routine part of clinical practice. In the pediatric rehabilitation course, observations were completed on 60 participants. As can be seen in Table 2, 78% independently determined functional problems during clinical observations, indicating successful transfer of didactic knowledge to clinical practice.

**TABLE 2.** Participants’ Ability to Determine Functional Problems During Structured Clinical Observations, N=60

**Performed Independently**	**Performed with Assistance**	**Not Performed**
47 (78%)	13 (22%)	0

Clinical observations indicated successful transfer of didactic knowledge to clinical practice.

### Course Assessments

Before this project, therapists did not routinely establish or progress functional goals. Instead the typical practice was to write general goals based on symptoms such as “reduce pain” or “improve strength.” As part of the pediatric course assessment (Supplement 2), participants were asked to rate their perceived competency in various indicators related to functioning (Table 3). As can be seen, 92% of respondents stated that they felt either “quite” or “very” confident in their abilities to establish functional goals and 97% stated that they were either “quite” or “very” confident in “progressing functional, meaningful treatment activities.”

**TABLE 3.** Perceived Competency in Various Indicators Related to Functioning at the Conclusion of the Pediatric Rehabilitation Course

**Thinking about the last 5 patients you saw in your workplace last week, how confident were in you in:**					
	**Very Much**	**Quite a Bit**	**Somewhat**	**A Little Bit**	**Not at All**
Identifying activity limitations? (N=63)^a^	53 (84%)	9 (14%)	1 (2%)	0	0
Establishing functional goals? (N=62)	28 (45%)	29 (47%)	5 (8%)	0	0
Selecting functional, meaningful treatment activities? (N=55)	28 (51%)	26 (47%)	1 (2%)	0	0
Progressing functional, meaningful treatment activities? (N=66)	29 (44%)	35 (53%)	1 (2%)	1 (2%)	0

a Not all participants answered every question resulting in a variation in the number of respondents.

### End-of-Grant Assessments

Assessments were distributed at the closing ceremonies of the project (Supplement 3). Fifty-five participants who attended at least one of the ARRSP courses completed the assessments. This represents 35% of the total number of unique course participants. As part of the assessment, they were asked to rate their perceived improvement in various aspects of patient-centered practice and clinical reasoning (Table 4). A Likert scale was utilized to measure improvement as follows: 0 = not at all, 5= somewhat, and 10 = a great deal. As can be seen in the table, the mode of perceived improvement in all indicators was 7 or greater on the Likert scale.

**TABLE 4.** Self-Rated Improvement in Patient-Centered Practice Measured at the End of the Grant Period

As a result of attending the Advancement of the Rwandan Rehabilitation Services courses, how much do you feel your evaluation and treatment of patients has improved for the following: (N=55) (Likert 0–10 Scale used)		
**Responses**	**Mode**	**Mean (SD)**
Using outcome measurements	7	7.8 (1.4)
Adjusting treatment based on patient improvement	9	8.2 (1.1)
Evaluating functional activities	8	8.4 (1.1)
Setting functional improvement goals	8	8.3 (1.2)
Clinical decision making	8	7.9 (1.1)

Abbreviation: SD, standard deviation.

### End-of-Grant Interviews

Near the end of the grant period, in-depth interviews were held with physiotherapy department managers from 4 major hospitals who had participated in ARRSP courses. Because it was not feasible to interview every course participant, we chose managers since they could offer insight to practice changes of therapists who worked in their departments. A written semistructured interview guide was used and responses were simultaneously recorded in writing. All responses to questions about outcome measures were positive. Below is a typical sampling of responses:

*We never used functional outcome scales previously. We now use the patient-specific functional scale. It helps us in formulating goals: Now we focus more on needs of the patient rather than just the physiotherapist’s expectations. Using the PSFS has also helped us with our clinical decision making. If a patient is progressing, we continue with the same treatment, but if they are not, then we change the treatment.* —Respondent

*The staff now uses the PSFS to help assess patients and form goals. We now measure goals more quantitatively, like timing how long a patient can stand.* —Respondent

*The physios now use the PSFS with each initial evaluation. It helps with discharge planning and progression of the patient.* —Respondent

### Interviews and Survey Conducted 26 Months After the Conclusion of the ARRSP

In the summer of 2017, a postgrant evaluation was conducted 26 months after the conclusion of the last course offering. An online survey was sent to the 65 participants of the pediatric rehabilitation course (Supplement 4). Forty-three people (66%) completed the survey. As can be seen in Table 5, 70% of respondents stated that they now used the PSFS either “a lot” or “quite a bit.”

**TABLE 5.** Responses to a Follow-up Survey Conducted 26 Months After the Conclusion of the Grant

**You were taught the use of the outcome measure called the patient-specific functional scale (PSFS). How often do you use the PSFS? (N=43)**	
**Responses**	**No. (%)**
A lot	14 (33)
Quite a bit	16 (37)
A little bit	8 (19)
Not at all	5 (12)

Additionally, the survey asked participants to rate their perceived competency in the same indicators related to functioning that they self-rated in the course assessment that was filled out on the last day of the course. Table 6 compares responses given at these 2 points in time. There was no more than a 10% difference in perceived competency at the 2 points in time, suggesting good sustainability of skills and concepts learned.

**TABLE 6.** Comparison of Perceived Competency Measurements Taken Immediately After the Pediatric Rehabilitation Course and Taken 26 Months After the Conclusion of the Grant

**Thinking about the last 5 patients you saw in your workplace last week, how confident were in you in:**	**“Quite a Bit” or “Very Much”**			
	**Postcourse Assessment**		**26-Month Follow-up**	
	**Total responses**	**No. (%)**	**Total responses**	**No. (%)**
Identifying activity limitations?	63	62 (98)	43	39 (91)
Establishing functional goals?	62	57 (92)	43	37 (86)
Selecting functional, meaningful treatment activities?	54	53 (98)	43	38 (88)
Progressing functional, meaningful treatment activities?	64	62 (97)	43	35 (81)

The perceived competency 26 months after the grant end remained high, suggesting good sustainability of skills and concepts learned.

In 2016, the Rwanda Ministry of Health outlined new guidelines for data collection stating that it is essential to collect accurate data to track patient outcomes.^22^ Performance standards are required in all 42 district hospitals in the country to attain re-accreditation. Monitoring clinical outcomes is designated as critical to improving quality and safety of care.^23^

Although these changes are positive, there are still obstacles to the long-term adoption of PROMs. Interviews and clinical observations held 26 months after the conclusion of the ARRSP revealed that PROMs were not regularly completed. Two frequently cited reasons included lack of promotion of the measures by department managers and the feeling among therapists that they were too time-consuming.

Two further obstacles were noted at the institutional level. Although we supplied participants with a number of PSFS booklets for use in the workplace, there were barriers to producing more. Copiers were not available in physiotherapy departments and blank sheets of paper were often in short supply. Additionally, initial evaluations were often stored centrally within hospital records and not easily accessible to therapists after the evaluation had been completed. This storage issue created an obstacle to intermittent monitoring to assess changes in function and modify treatment programs accordingly.

## DISCUSSION

Results indicate that the use of the PSFS reached encouraging levels of acceptability, adoption, feasibility, and penetration at the end of the grant period. Additionally, participants demonstrated greater understanding and utilization of patient-centered practice techniques. The postgrant evaluation found that perceived confidence in establishing functional goals; selecting functional, meaningful treatment activities; and progressing those activities remained high 26 months after the grant period ended. However, the evaluation also revealed obstacles that limited optimal sustainability of the use of PROMs. Both institutional and clinical practice challenges were cited.

The use of the PSFS reached encouraging levels of acceptability, adoption, feasibility, and penetration at the end of the grant period.

**FIGURE 3.**
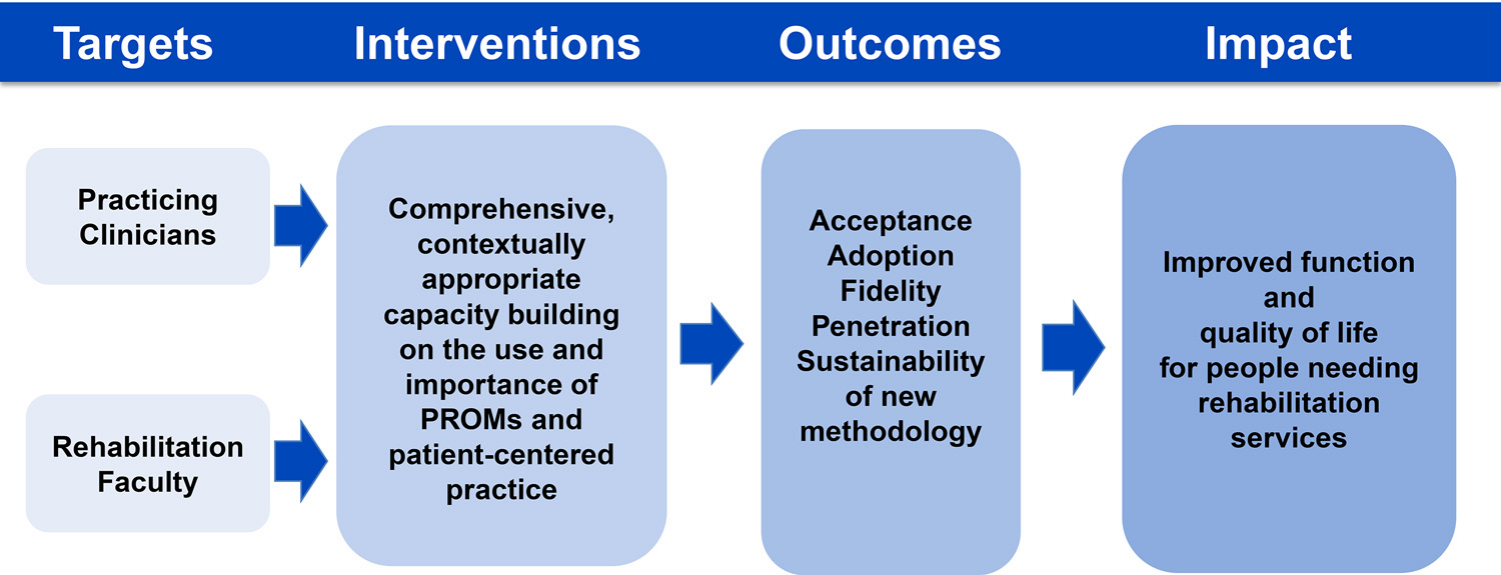
Capacity-Building Model Used to Train Physiotherapists in Rwanda

The challenges in achieving long-term sustainability highlight difficulties that can be encountered when introducing new methodologies in low-resource settings. There must be a solid foundation upon which to introduce new practice standards. Accomplishing this is difficult without support at every level of the health system. In this project, we used the capacity-building model illustrated in Figure 3 in which the targets for interventions were practicing clinicians and rehabilitation faculty.

**FIGURE 4.**
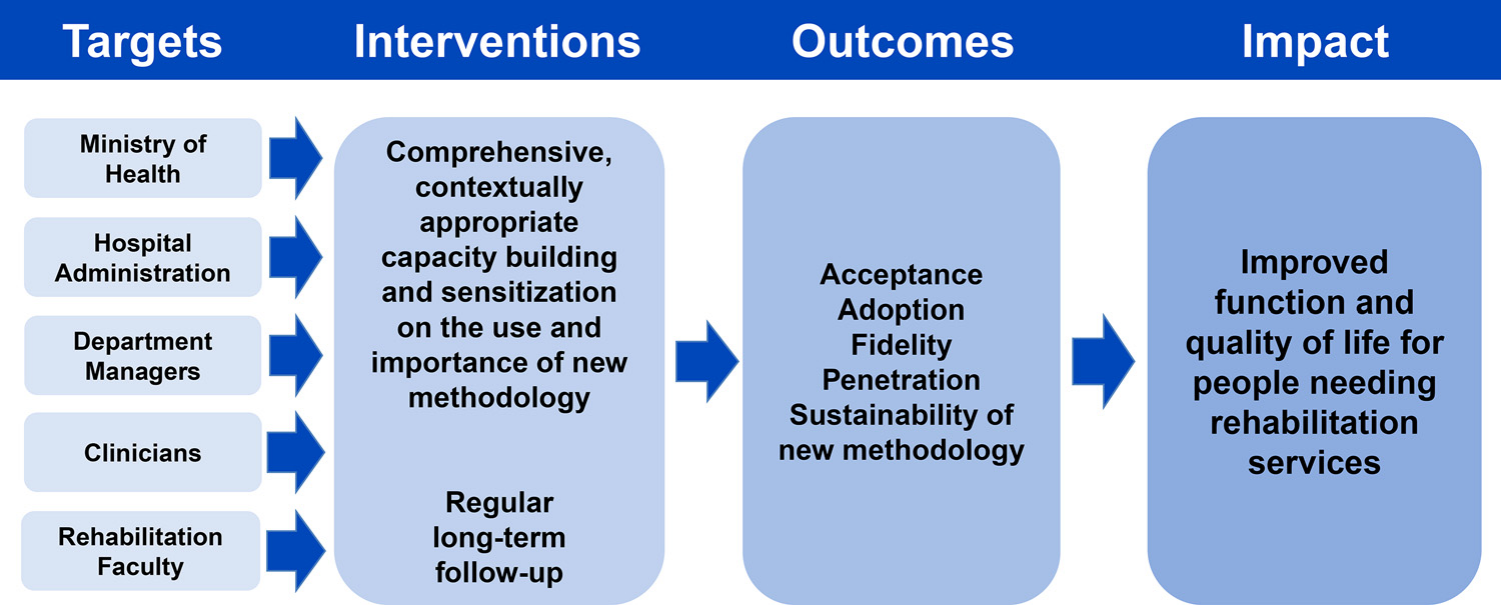
Capacity-Building Model That Should Have Been Used to Train Physiotherapists in Rwanda

In retrospect, we think the model in Figure 4 would have been better. By eliciting support from multiple levels of the health system including the Ministry of Health, hospital administration, and physiotherapy department managers, we believe that sustainability of new methodologies and practice techniques could have been enhanced.

### Limitations and Lessons Learned

Although the Rwandan steering committee requested that ARRSP courses emphasize clinical decision making, the majority of physiotherapists in Rwanda were not familiar with outcome measures and patient-centered practice. Therefore, there were uneven perceptions of need for change and motivation to change. Some therapists understood that using PROMs was an essential aspect of clinical decision making and were motivated to use them. But others did not fully integrate them into their practices.

Because the end-of-grant assessment was distributed at the closing ceremonies, it was only available to those in attendance, which was approximately 35% of course participants. If we could do it over, we would send out an electronic version of the assessment to reach more participants.

The postgrant assessment was performed 26 months after the grant closing and only included participants who attended the pediatric rehabilitation course. This was due to the interest, availability, and motivation of one of the Rwandan co-instructors in sampling this subset. In hindsight, we would have implemented the postgrant assessment 12 months after the final course and included participants from all ARRSP courses to assess the levels of success across all participants. Additionally, the pediatric group was challenging since many of the patients had long-term congenital conditions that do not respond quickly or completely to physical therapy interventions.

## CONCLUSION AND RECOMMENDATIONS

We recommend that groups planning similar capacity-building endeavors consider the model presented in Figure 4 to target stakeholders from a broad range of health system levels. We believe that this approach will better facilitate systemic and institutional support to maximize adoption, penetration, and sustainability of new skills and concepts. We hope that methodologies utilized and lessons learned from this study are useful to other medical professionals planning capacity training in low-resource settings.

## Supplementary Material

19-00408-Mann-Supplement_4.pdf

19-00408-Mann-Supplement_1.pdf

19-00408-Mann-Supplement_3.pdf

19-00408-Mann-Supplement_2.pdf

## Notes

**Peer Reviewed**

**First published online:** July 28, 2020
