# Supplementary material for: Using Patient-Reported Outcome Measures to Promote Patient-Centered Practice: Building Capacity Among Pediatric Physiotherapists in Rwanda
Source: Glob Health Sci Pract. 2020 Sep 30;8(3):596–605. doi: 10.9745/GHSP-D-19-00408 (PMC7541114; doi:10.9745/GHSP-D-19-00408)
Supplement: 19-00408-Mann-Supplement_4.pdf [file 19-00408-Mann-Supplement_4.pdf]

19-00408-Mann-Supplement 4
Supplement to: Mann M, Musabyemariya I, Harding L, Braxley B. Promoting patient-centered practice through the use of patient reported outcome measures: building capacity among pediatric physiotherapists in Rwanda. *Glob Health Sci Pract*. 2020;8(3). https://doi.org/10.9745/GHSP-D-19-00408

ARRSP Pediatric Rehabilitation: 26 Month Postgrant Survey

CONSENT FORM
I agree to participate in this evaluation of Advancement of Rwandan Rehabilitation Project: Pediatric Rehabilitation Course
Yes
No

What is your age?
Between 20 and 30
Between 31 and 40
Between 41 and 50
Between 51 and 60

What is your sex?
Female
Male

Level of education
Master's Degree
Bachelor's Degree
Advanced Diploma
Other - Write In

In which setting do you work from? (Check all that apply)
University Teaching Hospital
Referral Hospital
Specialized hospital (Gatagara Orthopedic Hospital, Rilima Orthopedic Hospital, Inkurunziza Orthopedic Hospital, others)
District hospital
Private clinic
Rehabilitation center
Other - Write In

In which area of the country is your workplace?
Kigali City
Eastern Province
Western Province
Northern Province
Southern Province
Other - Write In

What responsibility do you have at your current job? (Check all that apply)
Head of Physiotherapy department
A physiotherapist

19-00408-Mann-Supplement 4
Supplement to: Mann M, Musabyemariya I, Harding L, Braxley B. Promoting patient-centered practice through the use of patient reported outcome measures: building capacity among pediatric physiotherapists in Rwanda. *Glob Health Sci Pract*. 2020;8(3). https://doi.org/10.9745/GHSP-D-19-00408

Lecturer/Assistant Lecturer
Other - Write In

How many years of experience do you have working as a physiotherapist?
1-2 years
3-5 years
6-10 years
More than 10 years

In my everyday work as a physiotherapist, pediatric cases usually make up about this amount of my patient load:
N/A (I do not see patients)
10 - 25%
26 - 50%
50-75%
More than 75%
100% (I only treat pediatric cases)

What was the most helpful part of the Pediatric Class?

What was the least helpful part of the Pediatric Class?

Has your treatment of pediatric patients changed based on what you learned in this course?
Not at all
A little bit
Quite a bit
Very much

Please give an example of how your practice has improved or state why it has not.

Please list three treatment ideas and/or techniques that you learned in the course.

Do you incorporate the ideas and techniques from the course into your practice?
Not at all
A little bit
Quite a bit
Very much

Please explain your answer.

Were the clinic visits that the course instructors made helpful to you?
Yes

19-00408-Mann-Supplement 4
Supplement to: Mann M, Musabyemariya I, Harding L, Braxley B. Promoting patient-centered practice through the use of patient reported outcome measures: building capacity among pediatric physiotherapists in Rwanda. *Glob Health Sci Pract*. 2020;8(3). https://doi.org/10.9745/GHSP-D-19-00408

No

Please explain:

How could the clinic visits be improved in future trainings?

You were taught the use of the Outcome Measure called the Patient Specific Functional Scale (PSFS). How often do you use the PSFS?
Not at all
A little bit
Quite a bit
A lot

Please give reasons why you don't use PSFS?

At what point in your treatment do you use PDFD with your patients?

Why do you perform the PSFS?

Thinking about the last five patient you saw in your workplace last week, how confident were you in:
Identifying activity limitations?
Identifying participant restrictions?
Establishing functional goals?
Selecting functional, meaningful treatment activities?
Progressing functional, meaningful treatment activities?
Teaching the family how to integrate activities at home?
Identifying assistive device or adaptive equipment needs?

During the course you received some toys to use at your work place. Have these toys been helpful?
Yes
No

Please explain and give examples.

What challenges do you meet now, in management of your pediatric clients?

19-00408-Mann-Supplement 4
Supplement to: Mann M, Musabyemariya I, Harding L, Braxley B. Promoting patient-centered practice through the use of patient reported outcome measures: building capacity among pediatric physiotherapists in Rwanda. *Glob Health Sci Pract*. 2020;8(3). https://doi.org/10.9745/GHSP-D-19-00408

What other subject would you like to learn about in future pediatric CPD courses?

ARRSP Pediatric Rehabilitation: 26 Month Post -Grant Survey

19-00408-Mann-Supplement 4
Supplement to: Mann M, Musabyemariya I, Harding L, Braxley B. Promoting patient-centered practice through the use of patient reported outcome measures: building capacity among pediatric physiotherapists in Rwanda. *Glob Health Sci Pract*. 2020;8(3). https://doi.org/10.9745/GHSP-D-19-00408

CONSENT FORM

I agree to participate in this evaluation of Advancement of Rwandan Rehabilitation Project: Pediatric Rehabilitation Course

Yes

No

What is your age?

Between 20 and 30

Between 31 and 40

Between 41 and 50

Between 51 and 60

What is your sex?

Female

Male

Level of education

Master's Degree

Bachelor's Degree

Advanced Diploma

Other - Write In

In which setting do you work from? (Check all that apply)

University Teaching Hospital

Referral Hospital

Specialized hospital (Gatagara Orthopedic Hospital, Rilima Orthopedic Hospital, Inkurunziza Orthopedic Hospital, others)

District hospital

Private clinic

Rehabilitation center

Other - Write In

In which area of the country is your workplace?

Kigali City

Eastern Province

Western Province

Northern Province

19-00408-Mann-Supplement 4
Supplement to: Mann M, Musabyemariya I, Harding L, Braxley B. Promoting patient-centered practice through the use of patient reported outcome measures: building capacity among pediatric physiotherapists in Rwanda. *Glob Health Sci Pract*. 2020;8(3). https://doi.org/10.9745/GHSP-D-19-00408

Southern Province
Other - Write In

What responsibility do you have at your current job? (Check all that apply)

Head of Physiotherapy department
A physiotherapist
Lecturer/Assistant Lecturer
Other - Write In

How many years of experience do you have working as a physiotherapist?

1-2 years
3-5 years
6-10 years
More than 10 years

In my everyday work as a physiotherapist, pediatric cases usually make up about this amount of my patient load:

N/A (I do not see patients)
10 - 25%
26 - 50%
50-75%
More than 75%
100% (I only treat pediatric cases)

What was the most helpful part of the Pediatric Class?

What was the least helpful part of the Pediatric Class?

Has your treatment of pediatric patients changed based on what you learned in this course?

Not at all
A little bit
Quite a bit
Very much

Please give an example of how your practice has improved or state why it has not.

Please list three treatment ideas and/or techniques that you learned in the course.

Do you incorporate the ideas and techniques from the course into your practice?

19-00408-Mann-Supplement 4
Supplement to: Mann M, Musabyemariya I, Harding L, Braxley B. Promoting patient-centered practice through the use of patient reported outcome measures: building capacity among pediatric physiotherapists in Rwanda. *Glob Health Sci Pract*. 2020;8(3). https://doi.org/10.9745/GHSP-D-19-00408

Not at all
A little bit
Quite a bit
Very much

Please explain your answer.

Were the clinic visits that the course instructors made helpful to you?

Yes
No

Please explain:

How could the clinic visits be improved in future trainings?

You were taught the use of the Outcome Measure called the Patient Specific Functional Scale (PSFS). How often do you use the PSFS?

Not at all
A little bit
Quite a bit
A lot

Please give reasons why you don't use PSFS?

At what point in your treatment do you use PSFS with your patients?

Why do you perform the PSFS?

Thinking about the last five patients you saw in your workplace last week, how confident were in you in:

Identifying activity limitations?
Identifying participation restrictions?
Establishing functional goals?
Selecting functional, meaningful treatment activities?
Progressing functional, meaningful treatment activities?
Teaching the family how to integrate activities at home?
Identifying assistive device or adaptive equipment needs?

During the course you received some toys to use at your work place. Have these toys been helpful?

Yes
No

19-00408-Mann-Supplement 4
Supplement to: Mann M, Musabyemariya I, Harding L, Braxley B. Promoting patient-centered practice through the use of patient reported outcome measures: building capacity among pediatric physiotherapists in Rwanda. *Glob Health Sci Pract*. 2020;8(3). https://doi.org/10.9745/GHSP-D-19-00408

Please explain why or why not and give examples.

During the course you were shown how to make cheap toys.
Have you used those toys in your practice or made toys since the training?

Yes
No

Please explain and give examples.

What challenges do you meet now, in management of your pediatric clients?

What other subject would you like to learn about in future pediatric CPD courses?
