# Supplementary material for: Using Patient-Reported Outcome Measures to Promote Patient-Centered Practice: Building Capacity Among Pediatric Physiotherapists in Rwanda
Source: Glob Health Sci Pract. 2020 Sep 30;8(3):596–605. doi: 10.9745/GHSP-D-19-00408 (PMC7541114; doi:10.9745/GHSP-D-19-00408)
Supplement: 19-00408-Mann-Supplement_1.pdf [file 19-00408-Mann-Supplement_1.pdf]

19-00408-Mann Supplement 1
Supplement to: Mann M, Musabyemariya I, Harding L, Braxley B. Promoting patient-centered practice through the use of patient reported outcome measures: building capacity among pediatric physiotherapists in Rwanda. *Glob Health Sci Pract*. 2020;8(3). https://doi.org/10.9745/GHSP-D-19-00408

Health Volunteers Overseas
Advancement of Rwandan Rehabilitation Services Project
Pediatric Rehabilitation Course Pre- or Post-test Assessment

DATE: ______________________
Pre or Post Course Assessment **(Circle one)**
Have you attended any of the previous ARRSP courses?
________ YES ________ NO
If yes, circle all classes attended:
Spine Extremities/Therapeutic Exercise Neurology
Type of Physiotherapy Degree:
____

All questions are multiple choice. Circle ONE answer for each question.

1. Using the Patient Specific Functional Scale (PSFS) with our pediatric patients and their families can help us:

    A. Perform a standardized, norm based assessment allowing us to compare our patient to other children his/her age.
    B. Understand and identify what activities are important to the child and his/her family.
    C. Make a diagnosis about what is wrong with the child.
    D. The information we learn from the PSFS does not really help us in our evaluation, choice of treatment or setting of goals for the child.

2. Which of the following answers is NOT considered a part of case management decision making?

    A. The treatment is functional and meaningful to the child and family.
    B. The child's family is at the center of the decision making process.
    C. Allowing the child's doctor to make all the decisions about what kind of treatment to provide.
    D. When making decisions about which treatment to provide, you should consider research evidence, as well as your own clinical expertise.

3. Children with disabilities learning new motor skills:

    A. Should never be allowed to make mistakes when they are learning new motor skills.
    B. Learn new motor skills best when you practice the same skill over and over without changing or varying it.
    C. You should never vary the amount of feedback you give these children.

Supplement to: Mann M, Musabyemariya I, Harding L, Braxley B. Promoting patient-centered practice through the use of patient reported outcome measures: building capacity among pediatric physiotherapists in Rwanda. *Glob Health Sci Pract*. 2020;8(3). https://doi.org/10.9745/GHSP-D-19-00408

D. Should be provided opportunities to practice the new motor skill many times in a variety of ways both in your treatment session and in a home program.

4. Which of the following is the more advanced gross motor skill for an infant under 1 year old?

   A. Brings hands to midline in supine
   B. Head control during sitting with support
   C. Rolls from supine to prone with head leading, trunk rotation
   D. Lifts head 45-90 degrees when in prone

5. Which of the following is NOT true about gross motor development and motor milestones?

   A. Motor milestones are not absolute; children may develop motor skills at different rates and times.
   B. Gross motor development occurs in a distal to proximal fashion; in other words, you should expect a child to have hip control before they have head control.
   C. When a baby is first born, flexion dominates their postures and movements, followed by extension postures and movements.
   D. Exploration of the environment is an important part of motor milestone progression.

6. Which answer would suggest the child might have a developmental delay?

   A. A ten month old who is not walking.
   B. A five month old who cannot sit without help.
   C. A nine month old who cannot roll.
   D. A four month old who cannot crawl.

7. You place your 5 month old patient on their back on the mat and notice her legs positioned in a frog legged position, her head turned to one side and her arms on the mat alongside her body. You would anticipate this baby to have:

   A. Hypertonia
   B. Good head control in supported sitting
   C. Tightness and contractures around her joints
   D. Hypotonia

8. Which of the following is a correct statement concerning a baby with hypotonia?

   A. A baby with hypotonia will demonstrate early and strong head control

Supplement to: Mann M, Musabyemariya I, Harding L, Braxley B. Promoting patient-centered practice through the use of patient reported outcome measures: building capacity among pediatric physiotherapists in Rwanda. *Glob Health Sci Pract*. 2020;8(3). https://doi.org/10.9745/GHSP-D-19-00408

B. A baby with hypotonia will need more assistance with sitting and rolling.
C. A baby with hypotonia will be a very active baby- kicking arms and legs often.
D. A baby with hypotonia is likely to have contractures.

9. Which of the following statements is NOT true about tone and spasticity:

A. Hypertonia and spasticity is the same thing.
B. Spasticity can fluctuate in children who have cerebral palsy.
C. Examination of tone should include both passive and active ROM.
D. For children who have cerebral palsy, spasticity predominates in antigravity muscles; flexors of the upper extremities and extensors of the lower extremities.

10. Which of the following statements is NOT true about the origin and pathophysiology of cerebral palsy?

A. An event that causes cerebral palsy can occur before a baby is born (prenatal), during the birth process (perinatal) or after a baby is born (postnatal).
B. For most children diagnosed with cerebral palsy, the brain damage occurred during the prenatal period from hypoxic-ischemic events.
C. Cerebral palsy can also be caused by infections or traumatic events to the mother.
D. Improving the prenatal health of mothers will not help reduce the incidence of cerebral palsy.

11. The Gross Motor Function Classification Scale (GMFCS) is:

A. Is used to classify children with autism.
B. Classifies children with cerebral palsy as mild, moderate or severe.
C. **Is a five level classification system based on functional abilities of children who have cerebral palsy**.
D. Can only be used for children with cerebral palsy who can walk.

Jean is a 10 month old boy brought to your clinic by his mother because she has several concerns about his motor development. His legs are stiff and he cannot sit or roll without help. When placed on the floor to play with his older brother, he falls over and cries. Answer the following three questions using the case study and the ICF model.

12. Based on the case study of Jean and the ICF model, which of the following is a Body Structure/Body Function Impairment?

A. Jean cannot sit without help.

Supplement to: Mann M, Musabyemariya I, Harding L, Braxley B. Promoting patient-centered practice through the use of patient reported outcome measures: building capacity among pediatric physiotherapists in Rwanda. *Glob Health Sci Pract*. 2020;8(3). https://doi.org/10.9745/GHSP-D-19-00408

B. Jean cannot roll without help.
C. Jean cannot sit on the floor to play with his brother.
D. Jean has stiffness in his legs.

13. Based on the case study of Jean and the ICF model, which of the following is an Activity Limitation?

A. Jean has stiffness in his legs.
B. Jean cannot sit on the floor to play with his brother.
C. Jean cries when he falls over.
D. Jean cannot sit without help.

14. Based on the case study of Jean and the ICF model, which of the following is a Participation Restriction?

A. Jean cannot roll without help.
B. Jean has stiffness in his legs.
C. Jean cannot sit on the floor to play with his brother.
D. Jean cannot sit without help.

15. Sensory integration treatment focuses on which three sensory systems?

A. Tactile, auditory, and visual
B. Tactile, proprioceptive, and vestibular
C. Tactile, auditory, and olfactory
D. Gustatory, auditory, and visual

16. A child who appears tired, has poor attention, likes to spin in circles and hang upside down, grinds his/her teeth, and cannot sit still can be classified with which type of sensory modulation disorder?

A. Under-arousal
B. Over-arousal
C. Sensory avoiding behavior
D. Over-stimulated

17. A child who does not like to be touched, avoids certain smells, has difficulty with new activities and changing activities, becomes easily frustrated, and prefers to sit alone and not play can be classified with which type of sensory modulation disorder?

A. Sensory seeking behavior
B. Under-arousal
C. Over-arousal
D. Autism

Supplement to: Mann M, Musabyemariya I, Harding L, Braxley B. Promoting patient-centered practice through the use of patient reported outcome measures: building capacity among pediatric physiotherapists in Rwanda. *Glob Health Sci Pract*. 2020;8(3). https://doi.org/10.9745/GHSP-D-19-00408

18. Which types of sensory treatment can calm a child who is overstimulated or over-aroused?

    A. Fast, arrhythmic swinging
    B. Spinning or twirling around
    C. Deep pressure
    D. Balancing on one foot

19. The best time to provide treatment to a baby in the NICU is during which behavioral state?

    A. Sleep- quiet or active
    B. Drowsy
    C. Quiet Alert
    D. Active Alert

20. Which of the following is NOT a stress sign in the neonate?

    A. Yawning
    B. Bringing hands together
    C. Splaying fingers
    D. Hiccupping or sneezing

21. Range of motion in the NICU:

    A. Should ONLY be performed by a physiotherapist
    B. Has been shown to increase bone density
    C. Must be performed 3-5x/day to be beneficial
    D. Can prevent developmental delay and help baby walk sooner
