# Supplementary material for: Using Patient-Reported Outcome Measures to Promote Patient-Centered Practice: Building Capacity Among Pediatric Physiotherapists in Rwanda
Source: Glob Health Sci Pract. 2020 Sep 30;8(3):596–605. doi: 10.9745/GHSP-D-19-00408 (PMC7541114; doi:10.9745/GHSP-D-19-00408)
Supplement: 19-00408-Mann-Supplement_3.pdf [file 19-00408-Mann-Supplement_3.pdf]

19-00408-Mann-Supplement 3

Supplement to: Mann M, Musabyemariya I, Harding L, Braxley B. Promoting patient-centered practice through the use of patient reported outcome measures: building capacity among pediatric physiotherapists in Rwanda. *Glob Health Sci Pract*. 2020;8(3). https://doi.org/10.9745/GHSP-D-19-00408

HEALTH VOLUNTEERS OVERSEAS ADVANCEMENT OF RWANDAN REHABILITATION SERVICES PROJECT
Final Evaluation

Which other ARRSP courses did you participate in? (please circle)

Spine	Therapeutic Exercise	Neurological Rehab	Pediatrics	Chest

Thinking about all of the ARRSP courses that you have participated in:

1. How much do you believe that your evaluation and treatment skills have improved in the following areas:
a. Clinical decision making
b. Use of active treatment technique
c. General Clinical skills
d. Directing treatment toward functional improvement
e. Use of Outcome Measurements

2. How much do you think awareness of physiotherapy has increased as a result of the

ARRSP courses?

3. What do you think needs to be done in order to continue to increase awareness of physiotherapy in Rwanda?

4. Please list three ways in which you have changed how you treat patients as a result of attending the ARRSP courses.

a.____

b. ____

c. ____

5. How have these changes benefitted your patients?

Supplement to: Mann M, Musabyemariya I, Harding L, Braxley B. Promoting patient-centered practice through the use of patient reported outcome measures: building capacity among pediatric physiotherapists in Rwanda. *Glob Health Sci Pract*. 2020;8(3). https://doi.org/10.9745/GHSP-D-19-00408

6. Have you heard any feedback from patients or other medical professionals regarding changes in your treatments? If so, please give examples.

7. Do you think that the standards of physiotherapy have improved as a result of the courses? How?

8. What do you think needs to be done to continue to improve the standard of physiotherapy in Rwanda?

9. Please explain how you think your Leadership Institute Project will affect the profession of physiotherapy in Rwanda?

10. What will help you in the completion of the project?

11. Would you attend CPD courses in the future if the expenses related to attending the course are not supported?

12. What topics would be useful for future CPD courses?

13. Do you have any other comments that you would like to share about the courses?
