# Supplementary material for: Using Patient-Reported Outcome Measures to Promote Patient-Centered Practice: Building Capacity Among Pediatric Physiotherapists in Rwanda
Source: Glob Health Sci Pract. 2020 Sep 30;8(3):596–605. doi: 10.9745/GHSP-D-19-00408 (PMC7541114; doi:10.9745/GHSP-D-19-00408)
Supplement: 19-00408-Mann-Supplement_2.pdf [file 19-00408-Mann-Supplement_2.pdf]

19-00408-Mann Supplement 2
Supplement to: Mann M, Musabyemariya I, Harding L, Braxley B. Promoting patient-centered practice through the use of patient reported outcome measures: building capacity among pediatric physiotherapists in Rwanda. *Glob Health Sci Pract*. 2020;8(3). https://doi.org/10.9745/GHSP-D-19-00408

Advancement of Rwandan Rehabilitation Services Project

Pediatric Rehabilitation Course Survey

Circle: Kigali A Kigali B Date:

How has your treatment of pediatric patients changed based on what you learned in this course? Please give three examples.

1.
2.
3.

List three **new** treatment skills or ideas that you learned in this course.

1.
2.
3.

Did the clinic visits help to reinforce the course content?

What benefits did you receive from having one of the Course Instructors visit your clinic?

How could the clinic visits be improved?

You were taught the use of Outcome Measure called the Patient Specific Functional Scale (PSFS). When would you perform this with your patients?

19-00408-Mann Supplement 2
Supplement to: Mann M, Musabyemariya I, Harding L, Braxley B. Promoting patient-centered practice through the use of patient reported outcome measures: building capacity among pediatric physiotherapists in Rwanda. *Glob Health Sci Pract*. 2020;8(3). https://doi.org/10.9745/GHSP-D-19-00408

What are some reasons for performing the PSFS?

- *Thinking about the last 5 patients you saw in your workplace last week, how confident were you in* **determining body structure and functional impairments**?

Not at all	A little bit	Somewhat	Quite a bit	Very much

- *Thinking about the last 5 patients you saw in your workplace last week, how confident were you in* **identifying activity limitations?**

Not at all	A little bit	Somewhat	Quite a bit	Very much

- *Thinking about the last 5 patients you saw in your workplace last week, how confident were you* **in identifying participation restrictions?**

Not at all	A little bit	Somewhat	Quite a bit	Very much

- *Thinking about the last 5 patients you saw in your workplace last week, how confident were you* **in establishing functional goals?**

Not at all	A little bit	Somewhat	Quite a bit	Very much

- *Thinking about the last 5 patients you saw in your workplace last week, how confident were you* **in selecting functional, meaningful treatment activities?**

Not at all	A little bit	Somewhat	Quite a bit	Very much

- *Thinking about the last 5 patients you saw in your workplace last week, how confident were you* **in progressing functional, meaningful treatment activities?**

Not at all	A little bit	Somewhat	Quite a bit	Very much

19-00408-Mann Supplement 2
Supplement to: Mann M, Musabyemariya I, Harding L, Braxley B. Promoting patient-centered practice through the use of patient reported outcome measures: building capacity among pediatric physiotherapists in Rwanda. *Glob Health Sci Pract*. 2020;8(3). https://doi.org/10.9745/GHSP-D-19-00408

- *Thinking about the last 5 patients you saw in your workplace last week, how confident were you in* **teaching the family how to integrate activities at home?**

Not at all	A little bit	Somewhat	Quite a bit	Very much

- *Thinking about the last 5 patients you saw in your workplace last week, how confident were you in identifying assistive device or adaptive equipment needs?*

Not at all	A little bit	Somewhat	Quite a bit	Very much
